# Optimizing RNAi-Target by *Nicotiana benthamiana*-Soybean Mosaic Virus System Drives Broad Resistance to Soybean Mosaic Virus in Soybean

**DOI:** 10.3389/fpls.2021.739971

**Published:** 2021-11-22

**Authors:** Hua Jiang, Kai Li, Junyi Gai

**Affiliations:** Soybean Research Institute & MARA National Center for Soybean Improvement & MARA Key Laboratory of Biology and Genetic Improvement of Soybean (General) & State Key Laboratory for Crop Genetics and Germplasm Enhancement & Jiangsu Collaborative Innovation Center for Modern Crop Production, Nanjing Agricultural University, Nanjing, China

**Keywords:** broad-spectrum resistance (BSR), host-induced gene silencing (HIGS), conserved target fragments (CTFs), soybean mosaic virus (SMV), transient assay in *N. benthamiana*, transgenic soybeans

## Abstract

Soybean mosaic virus (SMV) is a prevalent pathogen of soybean (*Glycine max*). Pyramiding multiple SMV-resistance genes into one individual is tedious and difficult, and even if successful, the obtained multiple resistance might be broken by pathogen mutation, while targeting viral genome *via* host-induced gene silencing (HIGS) has potential to explore broad-spectrum resistance (BSR) to SMV. We identified five conserved target fragments (CTFs) from *S1* to *S5* using multiple sequence alignment of 30 SMV genome sequences and assembled the corresponding target-inverted-repeat constructs (TIRs) from S1-TIR to S5-TIR. Since the inefficiency of soybean genetic transformation hinders the function verification of batch TIRs in SMV-resistance, the *Nicotiana benthamiana-*chimeric-SMV and *N. benthamiana-*pSMV-GUS pathosystems combined with *Agrobacterium*-mediated transient expression assays were invented and used to test the efficacy of these TIRs. From that, S1-TIR assembled from 462 bp CTF-*S1* with 92% conservation rate performed its best on inhibiting SMV multiplication. Accordingly, S1-TIR was transformed into SMV-susceptible soybean *NN1138-2*, the resistant-healthy transgenic T_1_-plants were then picked out *via* detached-leaf inoculation assay with the stock-plants continued for progeny reproduction (T_1_ dual-utilization). All the four T_3_ transgenic progenies showed immunity to all the inoculated 11 SMV strains under individual or mixed inoculation, achieving a strong BSR. Thus, optimizing target for HIGS *via* transient *N. benthamiana-*chimeric-SMV and *N. benthamiana-*pSMV-GUS assays is crucial to drive robust resistance to SMV in soybean and the transgenic S1-TIR-lines will be a potential breeding source for SMV control in field.

## Introduction

Soybean mosaic virus (SMV) is a prevalent viral pathogen of soybean [*Glycine max* (L.) Merr.], especially in China (Hill and Whitham, 2014; Li and Zhi, 2016); it usually leads to soybean yield reductions that varied with cultivar, virus strain, location, and infection time (Hajimorad et al., 2018). Soybean mosaic virus belongs to *Potyvirus*; its genome is a single-stranded, positive-sense (+) RNA, with a length of ∼ 9.6 Kb. It is prone to high mutation rates, generating genetic diversity and different SMV strains (Seo et al., 2009b). Based on the symptoms produced on a set of different soybean genotypes, SMV isolates can be classified into different strain groups. For instance, seven SMV strains were reported in the United States based on the responses of a set of eight differential soybean genotypes to the virus (Cho and Goodman, 1979). Similarly, five SMV strains in Japan and eleven SMV strains in Korea have been reported (Takahashi et al., 1980; Seo et al., 2009a). In China, more than 4,500 country-wide SMV isolates were collected and grouped into twenty-two strains based on the responses of ten differential hosts to the virus at the National Center for Soybean Improvement (Li et al., 2010; Wang et al., 2014).

For different SMV strains, a number of SMV resistance loci were identified in host soybean-resistant sources. For example, three loci, *Rsv1*, *Rsv3*, and *Rsv4* in the United States and sixteen loci, *Rsc3* ∼ *Rsc8*, *Rsc10* ∼ *Rsc15*, *Rsc17*, *Rsc18*, *Rsc20*, and *Rsc21* in China have been identified from different resistant sources (Li and Zhi, 2016; Karthikeyan et al., 2017). However, pyramiding resistance genes conferring all SMV strains in a region or country into one individual using conventional methods is difficult and tedious, and it is always accompanied with undesirable traits from multiple parental sources (Malav et al., 2016). Besides, the breakdown of *R* gene-mediated resistance occurred frequently due to high mutation rates of SMV genome, interactions between the virus and its host, and the strong directional selection pressure created by the wide use of limited resistant resources (Choi et al., 2005; Gagarinova et al., 2008). In improving the situation and obtaining broad-spectrum resistance (BSR) to SMV, we have attempted to solve the SMV-disease problem based on combining host-induced gene silencing (HIGS) strategy with genetic engineering technology.

Host-induced gene silencing or host-delivered RNAi is a natural antiviral mechanism, which is initiated when double-stranded RNAs or hairpin RNAs (hpRNAs) are processed by dsRNA-specific dicer-like enzymes into 21- to 24-nucleotide (nt) short/small interfering RNAs which are then incorporated into the RNA-induced silencing complex to guide the degradation or translational repression of RNA targets in a sequence-specific manner (Yang and Li, 2018). Indeed, the hpRNAs are formed by the expression of the target-inverted-repeat (TIR) sequence (“target” is a piece of sequence of the infectious agent served as the stem in the structure of hpRNAs) in the corresponding host plant in most cases. For example, the transgenic soybean plants containing *CP*-TIR sequences of *Soybean dwarf virus* were developed and shown to be resistant to *Soybean dwarf virus* (Tougou et al., 2006). The transgenic soybean plants containing *CP-* or *HC-Pro*-TIR sequences of SMV showed resistance to SMV, but only a few plants exhibited SMV resistance in T_1_ generation (Kim et al., 2013, 2016; Gao et al., 2015a) while most transgenic lines transformed with SMV-CP-TIR sequences were susceptible to SMV inoculation. Although *CP-* or *HC-Pro*-targeting transgenes may be effective to a limited range of SMV strains, HIGS has shown potentials in exploring BSR to SMV. A key step for the success of HIGS strategy may lie in the identification of suitable target fragments common in numerous viral genomes since a conserved common target may cope with SMV strains with the same target sequence. However, due to low efficiency, time-consumption, and labor-inefficiency of soybean genetic transformation, to find a quick and efficient system for optimizing the best one from a mass of potential targets of HIGS before soybean genetic transformation is a key for the development of BSR to SMV.

*Nicotiana benthamiana* is an ideal model plant for the investigation of plant–pathogen interaction mainly due to a large number of diverse plant viruses that can successfully infect it, and *Agrobacterium*-mediated transient gene expression works exceptionally well in *N. benthamiana* plants (Norkunas et al., 2018). It has been successfully used for the identification of new viral suppressors of RNA silencing and functional analysis of unidentified genes, artificial microRNAs, or RNAi constructs to different plant viruses (Thomas et al., 2003; Peart et al., 2005; Goodin et al., 2008; Roumi et al., 2012; Medinahernández et al., 2013). In general, *N. benthamiana* plants could not be infected by SMV except several reports (Almeida et al., 2002; Gao et al., 2015b; Jiang et al., 2017). Thereinto, our previous study found that *N. benthamiana* plants can be systemically infected by an SMV of *Watermelon mosaic virus* (WMV) chimeric isolate 4278-1 (SWCI-4278-1), which has certain potential to verify the effectiveness of TIR constructs derived from the conserved target fragment (CTF) (Medinahernández et al., 2013). In addition, a recombinant SMV vector, pSMV-GUS was also considered for testing the infectivity of *N. benthamiana* plants in this study. Thus, we expect to use the two *N. benthamiana–*SMV pathosystems combined with *Agrobacterium*-mediated transient expression assay to quickly and efficiently select the possible HIGS targets, which are to be further verified for their function using a limited scale of transformed soybeans.

Accordingly, to explore the utilization of HIGS for BSR to SMV in soybeans, this study was focused on the following factors: (i) identifying CTFs throughout SMV genomes based on multiple sequence alignment and assembling TIR constructs, (ii) establishing and demonstrating a transient assay for identifying optimal TIR constructs on inhibiting SMV-multiplication using *N. benthamiana–*SWCI-4278-1 and *N. benthamiana–*pSMV-GUS pathosystems, and (iii) establishing a transgenic demonstrating system with dual-utilization of T1 in identifying SMV-resistant positive transgenic individuals and in obtaining corresponding progenies for a large-scale testing of the resistance spectrum. Based on the study, the obtained BSR materials might be used in real breeding programs.

## Materials and Methods

### Plant Materials and Soybean Mosaic Virus Strains

The broad-spectrum susceptible host soybean cultivar *NN1138-2* was used throughout the study along with the transient assay host, *N. benthamiana.* The *NN1138-2* and *N. benthamiana* plants, grown at 25°C with a 16 h photoperiod in a greenhouse, were used for *Agrobacterium tumefaciens*-mediated transformation, agroinfiltration, and SMV inoculation. Altogether 11 SMV strains (SC1, SC2, SC3, SC4, SC6, SC10, SC13, SC16, SC17, SC18, and SC19) provided by the National Center for Soybean Improvement (Nanjing Agricultural University, Nanjing, China) were used in testing for resistance spectrum.

### Identification of Conserved Target Fragments of Soybean Mosaic Virus Genome and Construction of Target-Inverted-Repeat Constructs

Conserved sequences are identical or have similar sequences of nucleic acids or amino acids. Sequence conservation is positively related to sequence identity. Accordingly, multiple sequence alignment was conducted using ClustalW algorithm for the complete genome sequences of 30 SMV isolates downloaded from the NCBI database^1^ (Supplementary Table 1). Five CTFs (*S1*–*S5*) were selected according to sequence conservation in the sequence alignment result and the length of the CTFs approximately range from 300 to 500 bp. The sequence conservation of these fragments was analyzed using DNaSP software with conserved DNA regions module in the analysis option, i.e., sequence conservation was calculated by the ratio of the number of variant sites to the net number of analyzed sites (Rozas et al., 2017). Then the sequences of the five fragments were cloned separately into pHellsgate12 (Helliwell and Waterhouse, 2003) to obtain S1-TIR, S2-TIR, S3-TIR, S4-TIR, and S5-TIR constructs, respectively; at the same time, the *S1* fragment was also cloned into pB7GWIWG2(II) (Karimi et al., 2002) to obtain pB7GWIWG2(II)-*S1*) using gateway method. The vector pB7GWIWG2(II) contains a bar gene as the selectable marker gene (conferring resistance to herbicide glufosinate). The primers required for the construction of these vectors are listed in Supplementary Table 2. All cloned sequences were confirmed by resequencing.

### Efficacy Validation of Target-Inverted-Repeat Constructs Using Transient *Nicotiana benthamiana –*SWCI-4278-1 and *Nicotiana benthamiana –*pSMV-GUS Pathosystems

From the *A. tumefaciens*-infiltrating leaves of *N. benthamiana*, eight leaves were infiltrated by each construct in one replicate, half is used for mock inoculation, and half is used for virus inoculation, and three replicates were conducted. The constructs from S1-TIR to S5-TIR were transformed into *A. tumefaciens* strain, GV3101 through electroporation, and the infiltration method was performed according to the protocols described by Wydro et al. (2006) with some modification. Briefly, the transformed *A. tumefaciens* GV3101 was cultured in 4 ml of Luria-Bertan (LB) medium (50 μg/ml spectinomycin) at 200 rpm for approximately 34 h at 28°C. The cell cultures were centrifuged at 4,500 rpm for 5 min and then the tight pellets were gently re-suspended in 10 mM MgCl_2_ (CAS:7786-30-3, Macklin Biochemical, Shanghai, China). The suspension was diluted to OD600 = 0.5 with a solution containing a final concentration of 10 mM MgCl_2_, 10 mM of 2-(N-morpholino) ethanesulfonic acid (MES) (CAS:145224-94-8, Sigma, United States) pH 5.7, and 150 μM of acetosyringone (CAS:2478-38-8, Biosharp, Hefei, China), and then infiltrated into 4-week-old leaves of *N. benthamiana* with a 1 mL needleless syringe.

Two virus inoculums were used for the inoculation of *A. tumefaciens* infiltrated leaves, where one is an SMV-WMV chimeric isolate SWCI-4278-1 and the other one is a recombinant SMV vector pSMV-GUS, i.e., a full-length of 9,994 bp SMV cDNA clone with GUS inserted between the P1 and HC-Pro cistrons were cloned into pGreen vector backbone, which was provided by the Laboratory of Plant Virology, Nanjing Agricultural University. For the inoculation of SWCI-4278-1 (Jiang et al., 2017), the SWCI-infected leaves of *N. benthamiana* were homogenized in 0.01 M phosphate buffer (PH 7.4) in a grinder and the tissue homogenates were filtered with two layers of cheesecloth. Then, the *A. tumefaciens*-infiltrated leaves of *N. benthamiana* were mechanically inoculated with a filtered sap. For the inoculation of infectious clone pSMV-GUS, the coinfiltration of infectious clone pSMV-GUS and TIR constructs was performed, i.e., an equal volume of both Agrobacterium cultures OD600 = 1.0 were mixed before infiltration. The inoculated or infiltrated plants were grown in insect-free chambers with 16 h light/8-h dark cycles at 25°C.

For virus detection of the SWCI-4278-1 and pSMV-GUS inoculated leaves, the real-time fluorescence quantitative PCR (qRT-PCR), ELISA and β-glucuronidase (GUS) histochemical staining and GUS activity were performed. For qRT-PCR, total RNA was extracted from *N. benthamiana* leaves using RNA isolation kit (Tiangen, Beijing, China) at 3 and 5dpi, respectively, and the quality and concentration of RNA were checked using agarose gel electrophoresis and a spectrophotometer (Nanodrop 2000, Thermo Fisher Scientific, MA, United States). The first-strand complementary DNA (cDNA) was synthesized using PrimeScript™ RT reagent Kit (TAKARA, Dalian, China) and qRT-PCR was performed according to previously described protocols (Jiang et al., 2019). JHA125 and JHA17 primers were used for amplifying the SMV isolate 4278-1 and pSMV-GUS, respectively; *EF1a* and *PP2A* were used as internal controls in *N. benthamiana* plants (Liu et al., 2012), and the relative accumulation of viral content was calculated by normalizing the geometric mean of the two reference genes. Each experiment was of three biological repeats, where each repeat had three technical replicates. The relative quantification of viral RNA was analyzed by 2^–△△CT^ methods based on the amplification efficiencies (Livak and Schmittgen, 2001; Pfaffl, 2001). The above primer sequences are listed in Supplementary Table 3.

For ELISA assay at 3 and 5dpi, the SMV antiserum (polyclonal rabbit antibodies) of ELISA was purchased from ACD Inc. (cat #V094-R1, Beijing, China) and the instructions of the manufacturer were followed for the next steps.

For GUS-histochemical staining and activity at 5dpi, histochemical staining of *N. benthamiana* leaves was performed using 5-bromo-4-chloro-3-indolyl glucuronide (X-gluc) (CAS:114162-64-0, War biological Technology, Nanjing, China) as described by Jefferson (1987). For fluorometric assays, the plant tissues were homogenized in an extraction buffer [50 mM phosphate buffer (pH 7.0), 10 mM of beta-mercaptoethanol (CAS:60-24-2, Solarbio Science &Technology, Beijing, China), 10 mM EDTA (CAS:6381-92-6, Solarbio Science & Technology, Beijing, China), 0.1% (w/v) of sodium dodecyl sulfate (CAS: 151-21-3, Solarbio Science & Technology, Beijing, China), 0.1% (V/V) Triton X-100 (CAS:9002-93-1, Solarbio Science &Technology, Beijing, China)], and centrifuged at 12,000 g for 15 min at 4°C. Enzyme reactions were performed in 200 μl of extraction buffer containing 1 mM of 4-methylumbelliferyl-β-d-glucuronide (CAS:6815-91-4, Shanghai, China) at 37°C, and were stopped by the addition of 20 μl reaction buffer and 180 μl of 0.2 M Na_2_CO_3_ (CAS:497-19-8, Solarbio Science & Technology, Beijing, China). Protein concentrations were quantified using the method described by Bradford (1976). The 4-methylumbelliferone fluorescence was measured using a SpectraMax M5 (Molecular Devices, Sunnyvale, CA, United States). GUS activities were expressed in nanomoles of 4-methylumbelliferone released per minute per milligram total protein.

### Northern Blot Analysis, Reverse Transcription-Polymerase Chain Reaction, and Western Blot Analysis

Total RNA was extracted from *N. benthamiana* leaves infiltrated with *A. tumefaciens*, non-transgenic and transgenic soybean leaves, respectively, using the Trizol reagent (Cat no. 15596-026, Invitrogen, Carlsbad, CA, United States) following the instructions of the manufacturer. The RNA specimens were electrophoresed on 15% polyacrylamide/7 M urea gels and transferred to Hybond N^+^ membranes (Amersham) using a semidry blotter (Bio-rad, Hercules, CA, United States). The membranes were UV cross-linked in a HL-2000 HybriLinker™ (AnalytikJena: UVP) at 120,000 μJ/cm^2^ energy for 4 min and separately hybridized with five different digoxin-UTP-labeled RNA probes. The mouse anti-digoxin monoclonal antibody (1:10000, Jackson ImmunoResearch) and IRDye 800CW-conjugated goat (polyclonal) anti-mouse IgG (1:15000; H + L; LI-COR Biosciences) secondary antibodies were used to evaluate siRNA production. The membranes were visualized using an LICOR Odyssey scanner with excitation at 700 and 800 nm. For Reverse Transcription-Polymerase Chain Reaction (RT-PCR) assay, the 320 bp SMV *CP* and 1,812 bp *GUS* bands were amplified from pSMV-GUS-infiltrated *N. benthamiana* leaves. The Western blot analyses were performed according to previously described protocols and the size of CP protein is ∼ 30 kDa (Jiang et al., 2019).

### Off-Target Prediction of *S1* Sequences, Soybean Transformation, and Identification of Transgenic Soybean Plants

The off-target searching of *S1* sequences in soybean *Williams 82* transcript database was conducted by siRNA-Finder software (Lück et al., 2019). The pB7GWIWG2(II)-*S1* was transformed into *A. tumefaciens* strain EHA105 by electroporation; the *A. tumefaciens*-mediated soybean genetic transformation was carried out according to the method described by Li et al. (2017). Genomic PCR, LibertyLink^®^ strip detection (QuickStix™ Kit purchased from EnviroLogix Inc., cat #AS 013 LS, Portland, ME, United States), and Northern blot were used to confirm gene insertion into soybean genome and gene expression. In addition, 492 bp *S1*, 557 bp *Tubulin*, and 399 bp *bar* bands were amplified from transgenic soybean plants. The primer sequences are listed in Supplementary Table 3. The genomic PCR primers were listed in Supplementary Table 3. LibertyLink^®^ strip detection was performed according to the instructions of the manufacturer. The siRNAs produced from the supposed transgenic soybean plants were detected using the Northern Blot method in the present study.

### Virus Inoculation of Detached Leaves *in vitro* and Transgenic Soybean Plants

When the unifoliate leaves of non-transgenic and transgenic soybean plants were fully expanded, one unifoliate leaf of each plant was split into two along the main vein and half of the unifoliate leaf was used for SMV strain SC18 inoculation. The SC18-infected leaves of soybean were homogenized in 0.01 M phosphate buffer (PH 7.4) in a grinder and the tissue homogenates were filtered with two layers of cheesecloth. Then, half of the unifoliate leaf was mechanically inoculated by a paintbrush using the filtered sap with a small amount of 600-mesh carborundum powder (CAS:409-21-2, Sinopharm Chemical Reagent, Shanghai, China). The SMV-inoculated leaves were placed in sterile petri dishes containing a wet filter paper and further covered with a piece of wet sterile cotton in the area near the petiole. The plates were then wrapped in a parafilm and placed in a growth-chamber at 25°C under 16 h photoperiod culture. After 7 days, the leaves were used for SMV detection through ELISA. For inoculation of non-transgenic and transgenic soybean plants, the 11 SMV strains were separately increased and maintained on a highly susceptible soybean *NN1138-2* and the SMV-infected soybean leaves were homogenized in 0.01 M phosphate buffer (PH 7.4) in different grinders and the tissue homogenates were filtered with two layers of cheesecloth, respectively. Then, the fully developed unifoliate leaves of soybean plants were mechanically inoculated with the above-mentioned filtered sap, respectively. For the mix inoculation, the individual saps of the same volume were mixed for inoculation. The number of plants used for each inoculation treatment is 20. The inoculated plants were grown in insect-free chambers with 16 h light/8 h dark cycles at 25°C, which were further used for resistance evaluation. ELISA detection assay was also performed at 28 dpi as per the instructions of the manufacturer.

### Statistical Analyses

The ELISA and qRT-PCR data were analyzed for their means, SD, and single-factor analyses of variance using the SPSS software (version 18.0, SPSS Inc., Chicago, IL, United States). Duncan’s new multiple range tests were performed to determine any significant difference among various treatments using the significance level of α = 0.05.

The *S1* nucleotide sequences were obtained from SMV (taxid: 12222) nucleotide collection of NCBI database, which were used for retrieving the highly similar sequences in the NCBI database by BLASTN suite with default parameter values, and the SMV genome sequences with significant alignments (the sequence identity is greater than 80%) were selected.

## Results

### Identification of Conserved Target Fragments in Soybean Mosaic Virus Genome

Focusing on the assumption of selecting relatively conserved sequences of SMV genome as the target of HIGS may contribute to the success of BSR to SMV, the potential target sequences in the SMV genome were analyzed through multiple sequence alignment of the complete genome sequences of 30 SMV isolates (Supplementary Table 1). The five CTFs (named *S1* to *S5*) were identified and distributed at different positions on the SMV genome (Supplementary Figure 1A). The five CTFs, *S1* to *S5*, varied in length from 322 to 497 bp with relatively low G/C and high A/U content (Table 1). The sequence conservation rates were 92%, 84%, 83%, 83%, and 86%, respectively, and varied with numbers of the conservative and variable sites among the fragments (Table 1). Meanwhile, the sequence conservation rate among the 30 SMV genomes was only ∼28%, far lower than that of the selected CTFs. Furthermore, S1-TIR to S5-TIR assembled from CTF *S1 to S5* was separately transiently expressed in *N. benthamiana* leaves, and the siRNAs produced from these constructs were detected by Northern blotting with respective DIG-labeled RNA probes. Supplementary Figure 1B shows the production of the siRNAs derived from five different CTFs and indicates that all the five TIR constructs can be expressed normally in *N. benthamiana* plants.

**TABLE 1:** Characteristics of the identified five conserved target fragments in Soybean mosaic virus (SMV) genome based on multiple sequence alignment.

Fragment name	Location^†^	Fragment length (bp)	G + C content (%)^‡^	A + U content (%)^§^	Number of variable sites	Number of conserved sites	Sequence conservation (%)
*S1*	2595–3056	462	39	61	38	426	92
*S2*	5601–6013	413	45	55	65	348	84
*S3*	6930–7426	497	39	61	83	414	83
*S4*	8261–8582	322	42	58	54	268	83
*S5*	8919–9274	356	42	58	50	306	86

*^†^ The different nucleotide positions of the conserved target fragments (CTFs) on SMV genome.*

*^‡^ The proportion of guanine and cytosine nucleotides in total nucleotides of the different CTFs, respectively.*

*^§^ The proportion of adenine and uracil nucleotides in total nucleotides of the different CTFs, respectively.*

### Differential Inhibition of SWCI-4278-1 Accumulation by Five Target-Inverted-Repeat-Constructs in *Nicotiana benthamiana* Leaves

In a previous study, it was found that the *N. benthamiana* plants could be systemically infected by an SMV-WMV chimeric isolate, i.e., SWCI-4278-1 (Jiang et al., 2017). It is not known, whether *N. benthamiana*–SWCI-4278-1 pathosystem can be used to optimize TIR constructs for inhibiting SMV multiplication. Thus, the S1-TIR, S2-TIR, S3-TIR, S4-TIR, and S5-TIR constructs with vector control were transiently expressed in *N. benthamiana* leaves by agroinfiltration, and further mechanically inoculated with SWCI-4278-1. The response and virus content were surveyed for the inoculated leaves at 3- and 5-days post-inoculation (dpi), respectively. No symptoms were developed on the inoculated leaves. The qRT-PCR results of the isolate SWCI-4278-1 nucleic acid is shown in Table 2. The amount of viral RNA was ∼12.8%– 14.8%, ∼24.2%– 32.5%, ∼21.6%– 27.5%, ∼17.4%–20.4% and ∼14.8%–16.3% in *N. benthamiana* leaves for S1-TIR, S2-TIR, S3-TIR, S4-TIR, and S5-TIR constructs, respectively, compared to the vector control (Table 2, left side). The significance tests showed that the S1-TIR was similar to the S5-TIR and superior to other three TIRs in the inhibition of viral nucleic acid accumulation at 5dpi (Table 2, left side). Consistent with the qRT-PCR analysis, the ELISA results revealed that the viral coat protein accumulated at lower levels (an average of ∼14.7%–29.4% at 3dpi and ∼18.0%– 34.7% at 5dpi) in S1-TIR, S2-TIR, S3-TIR, S4-TIR, and S5-TIR-expressed leaves compared to the vector control expressed leaves (Table 3). It was especially true for S1-TIR and S5-TIR according to Duncan’s new multiple range tests (α = 0.05). Collectively, these data indicated that the *N. benthamiana–*SWCI-4278-1 pathosystem combined with *Agrobacterium*-mediated transient expression assay worked exceptionally well in the screening of high-efficient TIR constructs. Thus SWCI-4278-1 infection was significantly inhibited in the inoculated leaves of *N. benthamiana* due to the expressed five different TIR constructs and the inhibition effect of S1-TIR and S5-TIR were superior over the other three TIR constructs on virus accumulation.

**TABLE 2:** Relative quantification of viral RNA in *N. benthamiana* leaves for different TIR constructs.

Sample name	SWCI-4278-1 as inoculum			Mock RQ	pSMV-GUS as inoculum		
	RQ (mean ± SD)		Percentage^†^ 3dpi/5dpi (%)		RQ (mean ± SD)		Percentage ^†^ 3dpi/5dpi (%)
	3dpi	5dpi			3dpi	5dpi	
EV	1085.65 ± 114.57a	2165.11 ± 172.53a	–	1.00	748.76 ± 21.28a	1504 ± 36.47a	–
S1-TIR	161.15 ± 9.90c	277.10 ± 25.59d	14.8/12.8	1.06	30.22 ± 3.34b	71.45 ± 1.66d	4.0/4.8
S2-TIR	352.35 ± 35.44b	523.39 ± 69.69b	32.5/24.2	0.94	45.12 ± 2.95b	121.62 ± 4.89b	6.0/8.1
S3-TIR	299.25 ± 26.31b	467.37 ± 39.16b	27.5/21.6	1.16	39.93 ± 1.43b	103.72 ± 4.50bc	5.3/6.9
S4-TIR	188.47 ± 20.56c	440.84 ± 27.58bc	17.4/20.4	1.00	34.35 ± 3.05b	83.23 ± 9.13cd	4.6/5.5
S5-TIR	177.43 ± 19.48c	319.99 ± 32.46cd	16.3/14.8	1.01	33.53 ± 1.74b	76.55 ± 6.53cd	4.5/5.1

*The mean and SD were calculated from three replications.*

*Values in the same column followed by different letters are significantly different at p = 0.05 level. RQ: relative quantification; SD: standard deviation.*

*^†^ Percentage was determined by the RQ means of each TIR sample divided by the RQ means of EV sample at 3dpi and 5dpi, respectively.*

*SWCI: SMV/WMV-chimeric isolate; EV: empty vector; TIR: target-inverted-repeat; dpi: days post-inoculation; -: no data; S1-TIR to S5-TIR represent the 5 respective TIR constructs; Mock: 0.01 M phosphate buffer inoculation as control.*

**TABLE 3:** The virus content in *N. benthamiana* leaves from DAS-ELISA analysis for different TIR constructs.

Sample name	ELISA (mean ± SD)			
	Mock	SWCI-4278-1		
		3dpi	5dpi	Percentage ^†^ 3dpi/5dpi (%)
EV	0.11 ± 0.01	1.02 ± 0.13a	1.67 ± 0.14a	–
S1-TIR	0.10 ± 0.01	0.15 ± 0.02c	0.30 ± 0.02d	14.7/18.0
S2-TIR	0.11 ± 0.01	0.30 ± 0.04b	0.58 ± 0.04b	29.4/34.7
S3-TIR	0.12 ± 0.01	0.17 ± 0.02c	0.47 ± 0.04bc	16.7/28.1
S4-TIR	0.10 ± 0.01	0.16 ± 0.02c	0.44 ± 0.04c	15.7/26.3
S5-TIR	0.12 ± 0.01	0.15 ± 0.02c	0.31 ± 0.02d	14.7/18.6

*The mean and standard deviation were calculated from three replications.*

*Values in the same column followed by different letters are significantly different at p = 0.05 level. SD: standard deviation.*

*^†^ Percentage was determined by the ELISA mean of each TIR sample divided by the ELISA mean of EV sample at 3dpi and 5dpi, respectively.*

*SWCI: SMV/WMV-chimeric isolate; EV: empty vector; TIR: target-inverted-repeat; dpi: days post-inoculation; -: no data; S1-TIR to S5-TIR represent the TIR constructs; Mock: 0.01 M phosphate buffer inoculation as control.*

### Viral Multiplication Was Inhibited by Five Target-Inverted-Repeat Constructs *via* Transient *Nicotiana benthamiana–*pSMV-GUS Pathosystem

**FIGURE 1:**
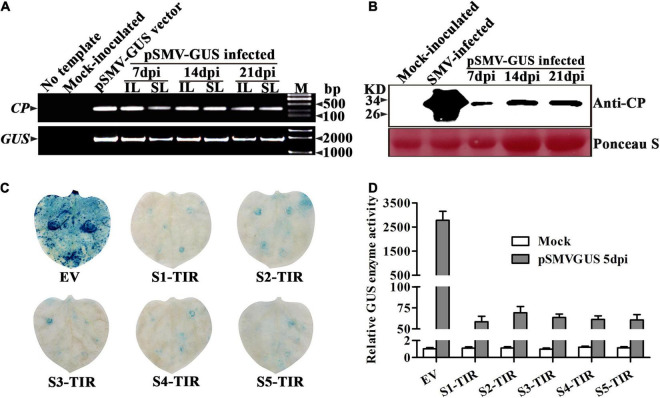
Systemic infection of recombinant SMV cDNA construct pSMV-GUS in *Nicotiana Benthamiana* plants and detection of pSMV-GUS accumulation in *N. benthamiana* leaves expressing S1-TIR to S5-TIR. **(A)** Detection of viral nucleic acid in inoculated and systemic leaves of *N. benthamiana* plants by RT-PCR. IL: inoculated leaves, SL: systemic leaves. **(B)** Western blot confirmed the presence of CP protein in the systemic leaves of *N. benthamiana* plants at 7, 14, and 21 dpi, respectively. **(C)** GUS histochemical staining of the inoculated leaves of *N. benthamiana* at 5 dpi. **(D)** GUS enzyme activity analysis of inoculated leaves infiltrated by different TIR constructs at 5 dpi. EV: empty vector, TIR: target-inverted-repeat, S1-TIR to S5-TIR represent the respective TIR constructs. Mock: 0.01 M phosphate buffer inoculation as control. The error bars indicate SD calculated from three biological replications.

For testing the infectivity of a recombinant vector pSMV-GUS in *N. benthamiana* plants, the phenotype, the viral nucleic acid, and coat protein of the pSMV-GUS infiltrated-*N. benthamiana* plants were assessed at 7, 14, and 21 dpi, respectively. No symptoms were observed on the inoculated leaves and in the non-inoculated systemic leaves. However, the RT-PCR analysis confirmed the existence of the viral nucleic acids in *N. benthamiana* plants infiltrated with pSMV-GUS using viral and GUS-specific primers, respectively, which indicates that the pSMV-GUS successfully replicated in the inoculated leaves and further spread to the non-inoculated leaves of *N. benthamiana* plants (Figure 1A). Meanwhile, the viral coat protein was detected in the systemic leaves of *N. benthamiana* plants by Western blotting, which confirmed the upward movement of pSMV-GUS in *N. benthamiana* plants (Figure 1B). Thus, *N. benthamiana* plants could be systemically infected by pSMV-GUS. Importantly, the friendly recombinant SMV infectious clone, pSMV-GUS can be used as an inoculum for testing the effect of the TIR constructs to SMV infection.

Next, whether pSMV-GUS infection can be restricted in *N. benthamiana* leaves by the five TIR constructs was investigated. *A. tumefaciens* containing individual different TIR constructs were mixed with the *A. tumefaciens*-contained pSMV-GUS in an equal proportion, respectively. The mixture was further infiltrated into *N. benthamiana* leaves through the injection method. As shown in Table 2, the relative content of pSMV-GUS nucleic acids was measured at 3dpi and 5dpi, respectively, using qRT-PCR. The amount of virus accumulation was ∼4.0%–4.8%, ∼6.0%–8.1%, ∼5.3%–6.9%, ∼4.6%–5.5%, and ∼4.5%–5.1% in *N. benthamiana* leaves for S1-TIR, S2-TIR, S3-TIR, S4-TIR, and S5-TIR constructs, respectively, compared to vector control-infiltrated leaves (Table 2, right side). The five TIR constructs performed similarly on the inhibition level of pSMV-GUS multiplication at 3 dpi, while the S1-TIR was superior to S2-TIR and S3-TIR and similar to S4-TIR and S5-TIR at 5dpi (Table 2, right side). Moreover, the GUS histochemical staining for all different inoculated leaves was observed at 5dpi (Figure 1C). As expected, the leaves coinfiltrated with vector control and pSMV-GUS displayed significantly higher GUS accumulation than the leaves coinfiltrated with each TIR construct and pSMV-GUS (Figure 1C), which indicated less virus accumulation in the five TIR constructs- expressed leaves. Meanwhile, the GUS enzyme activity test was in accordance with the observed histochemical analysis (Figure 1D). According to the above results, the efficacy of five TIR constructs on inhibiting virus multiplication was successfully validated based on transient *N. benthamiana*–pSMV-GUS pathosystem. From the above, the results of two transient *N. benthamiana*–SWCI-4278-1/pSMV-GUS assays both indicated that the CTF-*S1* and CTF-*S5* are more effective targets for *N. benthamiana*-induced gene silencing than the others, while between the two CTFs, CTF-*S1* should be more potential because of its higher sequence conservation (Figure 1 and Tables 1–3). Thus, the S1-TIR will be transformed into SMV-susceptible soybean, and the resistance of transgenic soybeans to SMV strains will be further evaluated.

### Generation of S1-TIR Transgenic Soybeans and Detached Leaf-Assay for Identifying Soybean Mosaic Virus Resistant T_1_-Plants

Before S1-TIR genetic transformation of soybean, the off-target searching of *S1* sequences in soybean *Williams 82* transcript database was conducted by siRNA-Finder software and no targets were found. The soybean genetic transformation assay obtained twelve independent T_0_ plants, which were transferred to the greenhouse, where one plant died and eleven survived. The S1-TIR insertion, bar protein existence, and siRNA production were analyzed for the randomly selected six transgenic T_0_ plants, and the results confirmed that the positive S1-TIR transgenic soybean plants were obtained (Supplementary Figure 2). All eleven transgenic T_0_ plants were self-fertilized to generate T_1_ generation.

**FIGURE 2:**
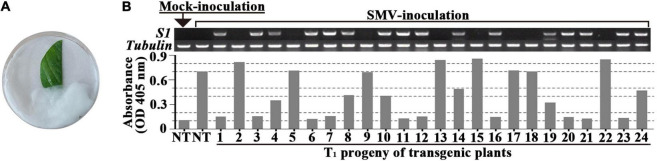
Evaluation of SMV accumulation in detached-leaves of T_1_ progeny of transformed soybean. **(A)** SMV inoculation and maintenance of the detached-leaf. **(B)** The genome PCR (upper side) and ELISA (down side) assay of the un-inoculated half of the unifoliate leaf at 7 dpi. The band size of *S1* fragment and *Tubulin* gene are 492 and 557 bp, respectively. NT: non-transgenic soybean; 1 to 24 are the individual transgenic T_1_-plants.

In order to screen out positive SMV-resistant individual plants from T_1_ generation and to avoid the potential effect of virus inoculation on transgenic plant growth and reproduction, one plant for two purposes (or dual functions) were adopted, i.e., SMV-resistance testing based on detached leaf-assay and the positive stock plants for generation extension and seed reproduction. The resistance-testing results of the randomly selected twenty-four T_1_ plants are graphically displayed in Figure 2. The virus content of half of a detached-unifoliate leaf inoculated with SMV SC18 sap was detected using ELISA at 7 dpi (Figure 2B, down side), and the other half of the detached-unifoliate leaf was used for S1-TIR construct insertion detection (Figure 2B, upper side). The results showed that the virus content is different among each detached-unifoliate leaf (Figure 2B). All T_1_ individual plants were tested based on the detached leaf-assay, and the positive rate and resistance rate of each transgenic line were counted (Table 4). In the T_1_ transgenic soybean plants, the positive rate was between 61.8 and 77.1%, and the resistance rate was between 28.3 and 60.5% (Table 4). The positive rate varied slightly while the resistance rate varied greatly. The results indicated that the differences in the proportion of resistant individual plants among different transgenic lines.

**TABLE 4:** Transgene-positive and resistant ratios in T_1_ generation from *in vitro* leaf-assay.

T_0_ code	Seed no.	No. of individual plants obtained	Transgene-positive plant		Resistant and susceptible plant^§^		R(%)^¶^
			No.^†^	(%)^‡^	(+)	(−)	
L1	100	89	60	67.4	43	17	28.3
L3	86	85	57	67.1	29	28	49.1
L4	17	17	11	64.7	6	5	45.5
L5	163	149	99	66.4	56	43	43.4
L6	255	223	172	77.1	68	104	60.5
L7	101	97	61	62.9	25	36	59.0
L8	65	60	39	65.0	20	19	48.7
L9	103	102	63	61.8	32	31	49.2
L10	28	23	17	73.9	7	10	58.8
L11	72	67	42	62.7	27	15	35.7
L12	108	92	57	62.0	27	30	52.6
Total	1098	1004	678	66.5	340	338	48.3

*^†^ The number of transgene-positive plants, identification of transgene-positive plants was based on genome DNA PCR using gene-specific primers.*

*^‡^ The percentage of obtained T_1_ plants.*

*^§^ Assessment of the resistant or susceptible plants was based on the ratio of the ELISA mean of each transgene-positive individual under SMV SC18 inoculation to the ELISA mean of negative control non-inoculated at 7dpi using in vitro leaf-assay. (+): positive for SMV (ratio ≥ 2); (-): negative for SMV (ratio < 2).*

*^¶^ The percentage of resistant plants.*

Moreover, the higher ratio was found in four transgenic lines (L6, L7, L10, and L12) (Table 4). Taken together, a total of 338 T1 SMV-resistant positive plants were obtained based on the detached leaf-assay. In brief, the developed approach is useful for preliminary screening SMV-strain resistance in individual plants.

### The S1-TIR Transgene Confers Robust Resistance to Multiple Soybean Mosaic Virus Strains in T_3_ Lines

**FIGURE 3:**
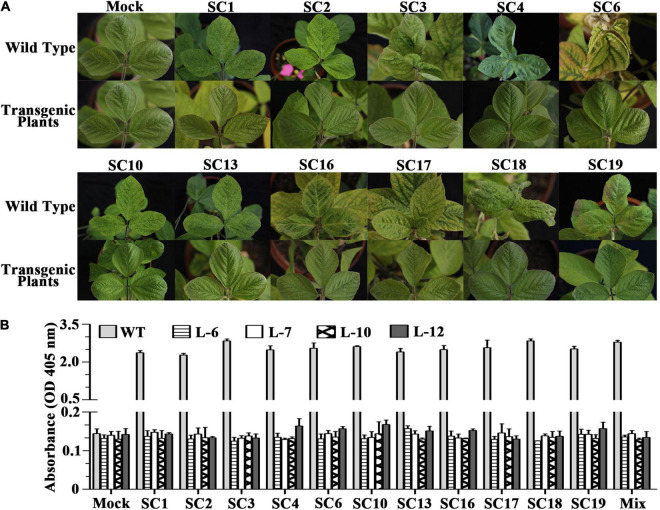
Responses of the non-transgenic and T_3_ transgenic soybean plants to SMV inoculation. **(A)** The photographs of the systemic leaves of SMV-inoculated non-transgenic and T_3_ transgenic plants were taken at 28 dpi. Wild Type = receptor soybean variety NN1138-2; Transgenic plants are those of Line L-6; Mock: non-inoculated plants as controls. SC1, SC2, SC3, SC4, SC6, SC10, SC13, SC16, SC17, SC18, and SC19: different SMV strains. **(B)** ELISA detection of SMV coat protein in the systemic leaves of SMV-inoculated non-transgenic (WT = NN1138-2) and four transgenic lines (L-6, L-7, L-10, and L-12) at 28 dpi. Mix: a mixed inoculum of the above eleven SMV strains. The error bars indicate standard deviations with *N* = 20.

In order to evaluate the performance of the whole plant of the transgenic progeny to multiple SMV strains, four T_3_ transgenic lines (L6, L7, L10, and L12) were selected and challenged with eleven SMV strains (SC1, SC2, SC3, SC4, SC6, SC10, SC13, SC16, SC17, SC18, and SC19), as control buffers and wild-type plants (susceptible soybean variety NN1138-2) were used. Figure 3 shows the responses of wild-type and transgenic plants to virus challenge. The typical symptoms of SMV include mosaic, mottling, and curling of soybean leaves, which may be different among strains. As shown in Figure 3A, the wild-type plants showed significant SMV symptoms on systemic leaves, while L6 transgenic plants showed a complete absence of symptoms for the eleven different SMV strains infection which was indistinguishable from the mock-inoculated controls at 28dpi (Supplementary Table 4). Similar results were also obtained for transgenic soybean plants L7, L10, and L12 after virus inoculation, confirming that SMV resistance can be seen in all four independent lines (Supplementary Table 4). Furthermore, the extracts of systemic leaves from individual plants were assayed by ELISA. As shown in Figure 3B, the SMV coat protein was undetectable in all transgenic plants inoculated with eleven SMV strains, confirming virus resistance.

Moreover, since the naturally mixed infection of SMV strains on soybean plants may occur in the field, T_3_ transgenic soybean plants were also tested with a mixed inoculum of eleven SMV strains (Figure 3B). No viral symptoms were detected on these four transgenic lines upon infectivity assay which were confirmed further by ELISA analysis (Figure 3B). Thus, the S1-TIR transgene conferred resistance to mixed infection of the eleven SMV strains in transgenic soybeans. Besides, the similarity analysis of *S1* among a wide range of SMV sequences showed that the identity ranged from 82.3 to 99.8% within SMV isolates from China, from 92.0 to 95.7% in Korea, from 92.0 to 97.8% with those from the United States, from 92.6 to 94.6% with those from Canada, from 93.5 to 97.6% with those from Iran, and from 82.8 to 95% with those from United Kingdom (Supplementary Table 5). It is especially important that the identity ranges from 91.8 to 99.8% in those SMV isolates collected from *Glycine* hosts, except several isolates collected from some atypical SMV hosts (Supplementary Table 5).

In brief, the CTF-*S1* obtained from SMV genome was demonstrated to be an efficient target of HIGS and did drive the development of BSR to SMV in soybean, so as to have a great potential in the breeding of novel soybean varieties with comprehensive resistance to various SMV strains or isolates and can benefit the field control of the virus.

## Discussion

### Identification of Conserved Target Fragment in Soybean Mosaic Virus Genome Is the Key for the Success of Host-Induced Gene Silencing-Mediated Broad-Spectrum Resistance

In this study, five CTFs for HIGS in SMV genome were identified based on the 30 SMV genome sequences, covering different source countries, with sequence conservation rate of 92%, 84%, 83%, 83%, and 86% for genome sequences from *S1* to *S5*, respectively. The results of transient *N. benthamiana*-SMV assays implied that the higher is the sequence conservation, the better is the working of HIGS. The higher sequence conservation of the CTF means it is more common among SMV genome sequences, and its TIR may recognize more extensive SMV strains and mediated BSR to SMV infection. Indeed, the CTF*-S1* not only showed very high nucleotide acid sequence identity (91.8 to 99.8%) with the complete genome sequences of about 50 strains/isolates isolated from cultivated and wild soybeans, but it also showed from 82.3 to 96.5% nucleotide acid sequence identity with the complete genome sequences of several SMV isolates collected from some atypical SMV hosts. It implies that S1-TIR might be of more potential in inducing BSR to more SMV strains or isolates with more effectiveness in the host and atypical hosts. That means the CTF-*S1* is a highly conservative segment among SMV genomes and S1-TIR might be potential in developing BSR to SMV.

On the other hand, the sequence conservation of a CTF may be related to its length and number of sequence pieces. The previous results showed that TIR constructs with different lengths of hairpin viral target sequence have been successfully used in various plant species (Das and Sherif, 2020). Both short (∼50 bp) and long (up to 2.5 Kbp) length of the stem in the hairpin structure were used (Lee et al., 2017). Integrating multiple small fragments with sequence conservation 100% as the CTF of HIGS might be a novel option for interference with more SMV strains, while it needs to be verified by experiments. In addition, the range of G/C content of our five CTFs was ∼39%–45%, meeting the criterion of 30%–52% G/C content (Fakhr et al., 2016). The highly G-rich sequences in the selection of siRNAs sequences should be avoided because they tend to form G-quartet structures. It explains why our five CTFs were all effective in using HIGS to induce the resistance to SMV, especially the BSR to SMV of CTF-*S1*.

### *Nicotiana benthamiana* –SWCI-4278-1/pSMV-GUS Pathosystems Form the Key for Rapid and Efficient Identification of Best Target-Inverted-Repeat Constructs

Several approaches for transiently identifying gene function in soybean and *N. benthamiana* have been reported in recent studies (Willman, 2015; Jiang et al., 2017, 2019; Wu and Hanzawa, 2018). For instance, a method of infiltrating soybean leaves with *Agrobacterium* was developed for transiently expressing DNA of interest, while the inadequacy of replicated experiments and the number of infectious tissues might have limited their practical implications (Willman, 2015). Wu and Hanzawa (2018) established a method for the isolation of soybean protoplasts and its application to transient gene expression studies, but this approach needs optimizing the conditions since many factors that affect protoplast yields and transformation rates were involved. Additionally, two pathosystems for studying soybean–virus interactions were developed, i.e., ARISHR-SMV (*Agrobacterium rhizogenes*-induced soybean hairy roots versus SMV) and *N. benthamiana–*SWCI-4278-1 pathosystems (Jiang et al., 2017, 2019). The former was demonstrated effective for at least nine SMV strains, while the latter takes less time for the functional analysis of unidentified genes since the transient expression in *N. benthamiana* leaves was rapid and reliable (Norkunas et al., 2018), and the leaves inoculated with the virus using mechanical friction were simple and convenient. In the present study, the virus accumulation was significantly inhibited in the leaves of *N. benthamiana* transiently expressed by five TIR constructs, and the inhibition effect of S1-TIR and S5-TIR were superior to others while S2-TIR showed the weakest inhibition ability. The effect comparison of different S1-TIR to S5-TIR constructs in virus inhibition using *N. benthamiana–*SWCI-4278-1 pathosystem confirmed that this approach is useful for identifying the best TIR construct.

Moreover, *N. benthamiana* plants can be systemically infected by a recombinant SMV infectious clone, i.e., pSMV-GUS; one possible reason is that extra 409 bp sequences of 5’ terminal region in SMV genome of pSMV-GUS is highly similar to that of *Bean common mosaic virus*, which also supported that the 5’-UTR and P1 region of the *Potyvirus* play important roles in the interaction between viruses and host (Zhang et al., 2015). Thus, the novel transient *N. benthamiana–*pSMV-GUS pathosystem can be also used for identifying the efficient target of HIGS. In comparison to SWCI-4278-1 as inoculum, the leaves agro-infiltrated with five TIR constructs accumulated less virus when using pSMV-GUS as inoculum, which is probably due to the fact that the viral replication level and/or the ability of HC-Pro suppressing posttranscriptional gene silencing were different between SWCI-4278-1 and pSMV-GUS. In brief, the transient *N. benthamiana–*SWCI-4278-1 and *N. benthamiana–*pSMV-GUS assays take only several days for optimizing the best TIR construct assembled from viral CTFs and the procedure was rapid and reliable. In contrast, if the effect verification of these TIR constructs to SMV was conducted by stable soybean genetic transformation, it will take a lot of time and workload. Thus, the advantage of saving time and effort will become more obvious when testing multiple TIR constructs using *N. benthamiana–*SMV pathosystem compared to the developing and testing of transgenic soybean plants. However, the reason that *N*. *benthamiana* can be infected by modified SMV (SWCI-4278-1 and pSMV-GUS) needs to be further explored.

### Dual-Utilization of Transgenic T_1_ Plants Is the Key for Goal-Directed Obtaining of Transgenic Resistant Individuals and Their Progenies

The detached leaf-assay has been used for evaluating the plant resistance to different pathogens (Aregbesola et al., 2020; Sun et al., 2020). In this study, for avoiding the influence of the inoculated SMV damaging to plant growth and reproduction, and the potential risk of passing the inoculated SMV to the next generation transgenic plants and for saving the workload of the transgenic seed reproduction, the detached leaf-assay was used for identifying SMV-resistant positive transgenic plants with the corresponding resistant stock plants used for seed reproduction. This dual utilization method of transgenic T_1_-plants is efficient and effective. This procedure may be used for rapidly screening transformed soybean materials at an early stage, which was used for filtering out the false positive plants and SMV-susceptible transgenic plants. Without this dual-utilization of T_1_-plants, many more transgenic plants have to be obtained and used in identifying their resistance. Of course, this procedure may have some issues out of expectation in SMV-resistance identification of non-transgenic soybean germplasm resources, such as the movement of the virus to the system leaves may be blocked in some soybean varieties, where even the inoculated-leaves can be infected by SMV (Wu and Cheng, 2020; Kumar and Dasgupta, 2021). In this case, the results of SMV resistance from the detached-leaves might appear false negative. In addition, In the T_1_ transgenic soybean plants, the positive rate varied slightly while the resistance rate varied greatly, which might be due to the insertion site or the expression level differences of S1-TIR among individuals. Anyway, the detached leaf-assay is still a rapid method to identify SMV resistance in soybean.

### The Integrated Host-Induced Gene Silencing Strategy Leads to Realizing the Broad-Spectrum Resistance to Soybean Mosaic Virus and Providing Novel Sources for Breeding Programs

Host-induced gene silencing has emerged as a powerful genetic tool in the fundamental research for the assessment of gene function and in applied research for plant protection and agronomic traits improvement, especially for viral control. In previous studies, most TIR-transgenic soybeans were susceptible to SMV inoculation; only a few plants exhibited SMV resistance in T_1_ generation (Kim et al., 2013). Similarly, Gao et al. (2015a) also reported that the TIR-transgenic soybean plants with resistance to a certain SMV strain was improved using partial SMV-*HC-Pro* sequences as the target for HIGS. Different from the previous studies, the present study revealed that all the four transgenic soybean lines that expressed the S1-TIR construct optimized via transient *N. benthamiana* assays were immune to all the inoculated 11 SMV strains. It implies that S1-TIR-induced resistance is really broad, strong, and stable. In short, the obtained 11 transgenic lines are of important value to be used in breeding programs coping with the SMV damage.

Recently, the clustered regulatory interspaced short palindromic repeats and associated protein system (CRISPR/Cas) were developed for editing specific-target genome sequences. Thereinto, it has been applied to different plants for resistance to virus infection, including cassava (Mehta et al., 2019), potato (Zhan et al., 2019), *N. benthamiana* (Aman et al., 2018), and so on. Some of them were able to successfully confer resistance to the target virus and others not. Moreover, a few research revealed that the CRISPR method with the potential to speed up virus evolution should be carefully assessed as they pose significant biosafety risks (Mehta et al., 2019). In contrast, HIGS-mediated resistance to the virus may be more efficient and environmentally safer (Ali et al., 2020; Das and Sherif, 2020). It seems to be a smart approach with a greater potential for developing the SMV-BSR transgenic soybeans because the action mechanism of HIGS enables it to target multiple SMV strains simultaneously, especially for adopting the CTF as the target of HIGS.

## Conclusion

In summary, CTF-*S1* as the target of HIGS was successful in developing BSR to SMV for soybean. In the integrated HIGS approach, high CTF conservation-rate, transient high-efficient verification of TIR *via N. benthamiana–*SWCI-4278-1 and *N. benthamiana–*pSMV-GUS pathosystems, and dual utilization of transgenic T_1_-plants are the key links in realizing BSR to SMV in soybean. In addition, the obtained transgenic S1-TIR-lines are novel potential sources for BSR to SMV in breeding for SMV-resistance.

## Data Availability Statement

The original contributions presented in the study are included in the article/Supplementary Material, further inquiries can be directed to the corresponding author.

## Author Contributions

JG and HJ conceived and designed the method and experiments. HJ and KL performed the experiments. HJ analyzed the data. JG contributed reagents, materials, and interpretation of the results. All authors read and approved the final manuscript.

## Conflict of Interest

The authors declare that the research was conducted in the absence of any commercial or financial relationships that could be construed as a potential conflict of interest.

## Publisher’s Note

All claims expressed in this article are solely those of the authors and do not necessarily represent those of their affiliated organizations, or those of the publisher, the editors and the reviewers. Any product that may be evaluated in this article, or claim that may be made by its manufacturer, is not guaranteed or endorsed by the publisher.

## Footnotes

1
http://www.ncbi.nlm.nih.gov/

## Supplementary Material

Supplementary Figure 1 The choice of SMV conserved fragments and verification of TIR constructs expression. **(A)** *S1* – *S5* distributed non-uniformly at different positions within SMV genome. *S1*: 2595 – 3056 nt, *S2*: 5601 – 6013 nt, *S3*: 6930 – 7426 nt, *S4*: 8261 – 8582 nt, and *S5*: 8919 – 9274 nt. **(B)** Expression of TIR constructs was verified in *N. benthamiana* leaves through transiently assay by Northern blot with different DIG-labeled RNA probes, respectively. EV: empty vector, S1-TIR to S5-TIR represent the TIR constructs.

Supplementary Figure 2 Identification of the positive transgenic soybean plants. **(A)** Genome DNA PCR amplification of the 492 bp *S1* and 399 bp *bar* gene in transformed soybean. -: ddH_2_O as a template; NT: non-transgenic soybean; +: vector control; L1, L3, L4, L5, L6, and L9 were T_0_ transgenic soybean lines. **(B)** LibertyLink^®^ strip detection. The first strip is the control line, and the second strip is the test line. **(C)** Northern blot analysis of siRNA isolated from T_0_ transgenic soybean lines (L1, L3, L4, L5, L6, and L9) detected by DIG-labeled S1 probe.
