# Supplementary figures and images for: Optimizing RNAi-Target by Nicotiana benthamiana-Soybean Mosaic Virus System Drives Broad Resistance to Soybean Mosaic Virus in Soybean
Source: Front Plant Sci. 2021 Nov 22;12:739971. doi: 10.3389/fpls.2021.739971 (PMC8645994; doi:10.3389/fpls.2021.739971)

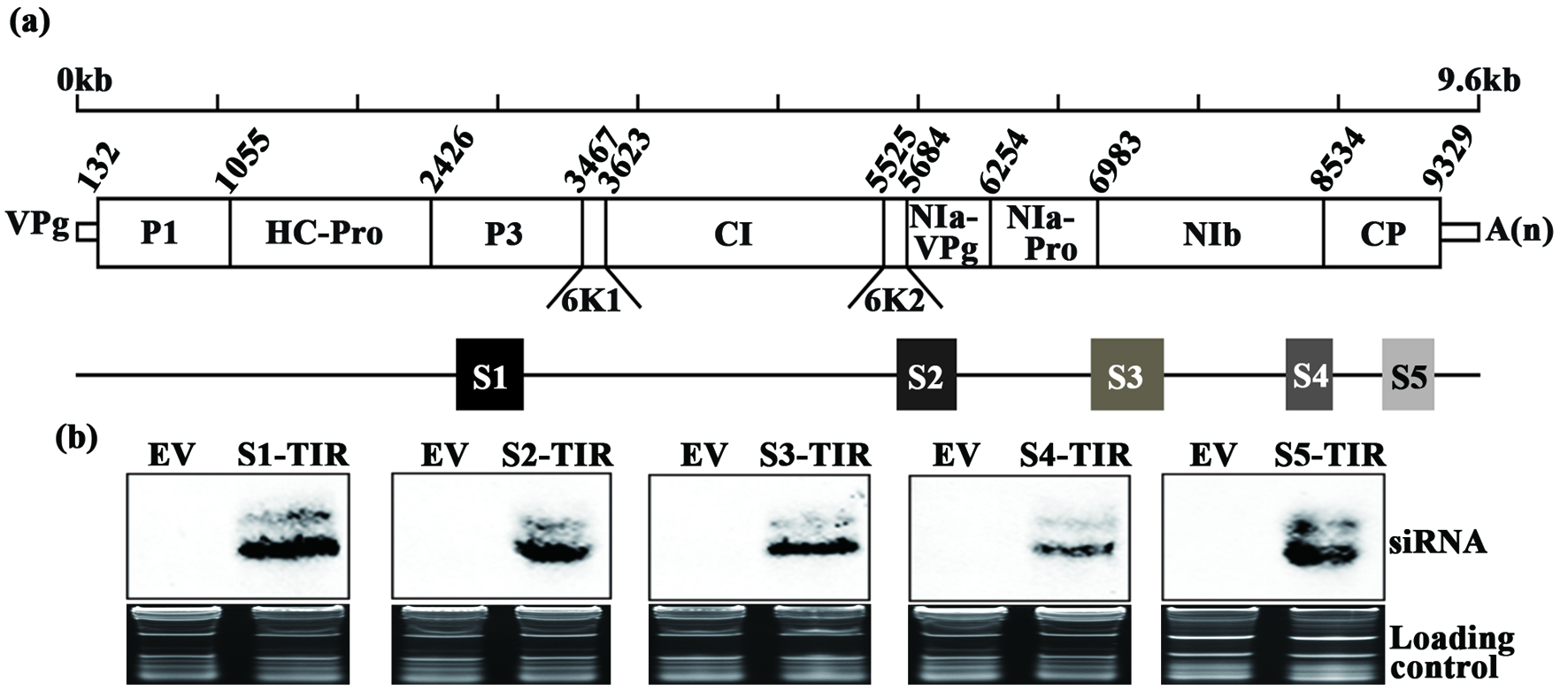

Supplement: Supplementary Figure 1 — The choice of SMV conserved fragments and verification of TIR constructs expression. (A) S1 – S5 distributed non-uniformly at different positions within SMV genome. S1: 2595 – 3056 nt, S2: 5601 – 6013 nt, S3: 6930 – 7426 nt, S4: 8261 – 8582 nt, and S5: 8919 – 9274 nt. (B) Expression of TIR constructs was verified in N. benthamiana leaves through transiently assay by Northern blot with different DIG-labeled RNA probes, respectively. EV: empty vector, S1-TIR to S5-TIR represent the TIR constructs. [file Image_1.TIF]

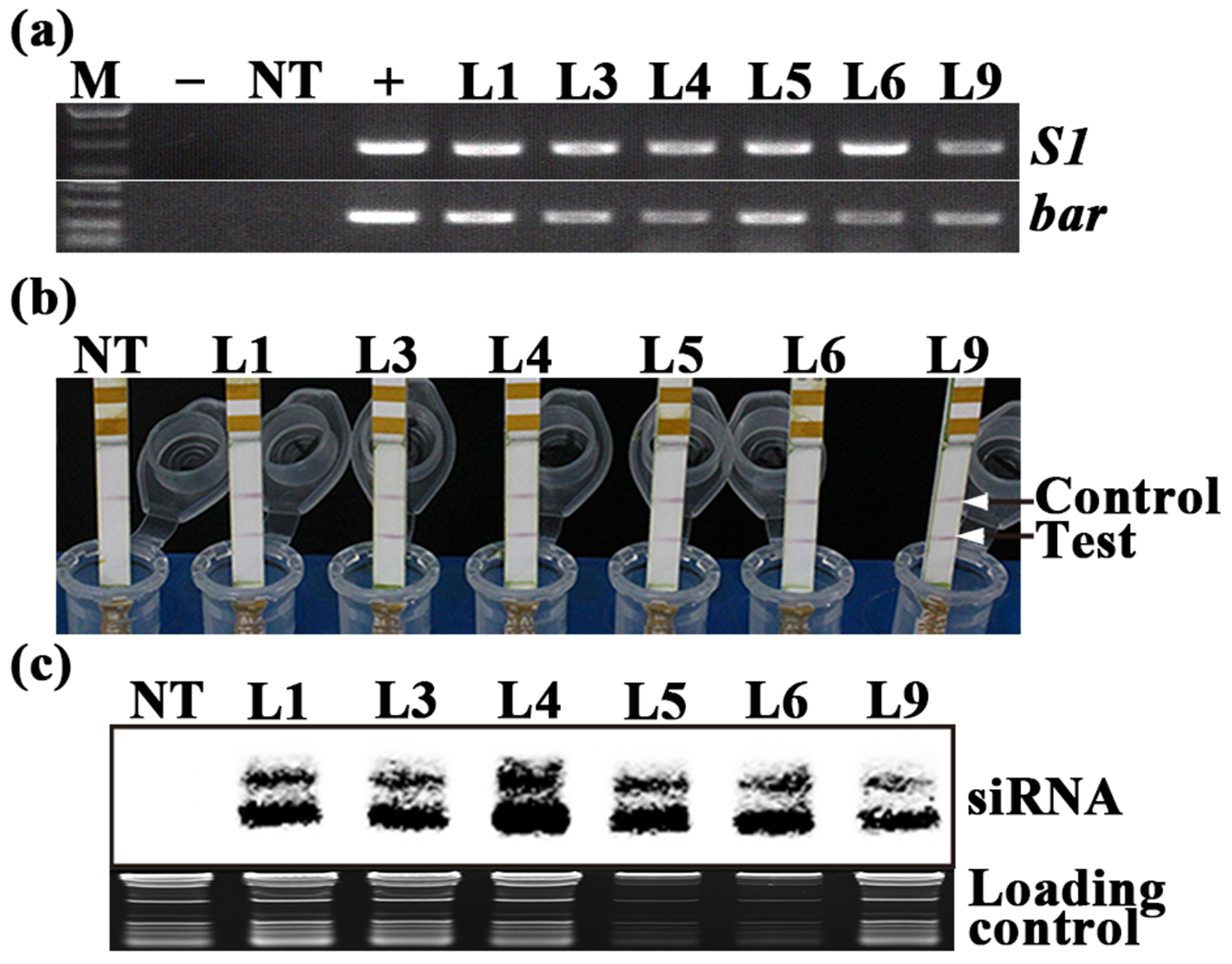

Supplement: Supplementary Figure 2 — Identification of the positive transgenic soybean plants. (A) Genome DNA PCR amplification of the 492 bp S1 and 399 bp bar gene in transformed soybean. -: ddH2O as a template; NT: non-transgenic soybean; +: vector control; L1, L3, L4, L5, L6, and L9 were T0 transgenic soybean lines. (B) LibertyLink® strip detection. The first strip is the control line, and the second strip is the test line. (C) Northern blot analysis of siRNA isolated from T0 transgenic soybean lines (L1, L3, L4, L5, L6, and L9) detected by DIG-labeled S1 probe. [file Image_2.TIF]
