# Supplementary material for: Optimizing RNAi-Target by Nicotiana benthamiana-Soybean Mosaic Virus System Drives Broad Resistance to Soybean Mosaic Virus in Soybean
Source: Front Plant Sci. 2021 Nov 22;12:739971. doi: 10.3389/fpls.2021.739971 (PMC8645994; doi:10.3389/fpls.2021.739971)
Supplement: Supplementary file 3 [file Table_1.DOCX]

**Table S1 The source of genomic sequences of 30 SMV isolates obtained from the NCBI database according to the GenBank ID.**

| NC_002634.1^‡^ | FJ640959.1^§^ | EU871724.1^\|\|^ | FJ640967.1^§^ | FJ640981.1^§^ | AB100443.1^¶^ |
| --- | --- | --- | --- | --- | --- |
| D00507.2^‡^ | FJ640976.1^§^ | FJ640979.1^§^ | AJ312439.1^†^ | FJ640980.1^§^ | AY294045.1^§^ |
| FJ640975.1^§^ | FJ640974.1^§^ | S42280.1^‡^ | FJ376388.1^§^ | FJ640957.1^§^ | FJ640971.1^§^ |
| FJ640972.1^§^ | FJ640966.1^§^ | FJ640955.1^§^ | FJ807701.1^§^ | FJ548849.1^§^ | FJ640954.1^§^ |
| FJ640968.1^§^ | EU871725.1^\|\|^ | FJ640963.1^§^ | FJ807700.1^§^ | AY294044.1^§^ | AB100442.1^¶^ |

Note: the different symbols represent the sequences of the isolate come from different countries, ^†^CHINA, ^‡^USA; ^§^KOREA, ^¶^JAPAN, ^||^CANADA
