# Supplementary material for: Optimizing RNAi-Target by Nicotiana benthamiana-Soybean Mosaic Virus System Drives Broad Resistance to Soybean Mosaic Virus in Soybean
Source: Front Plant Sci. 2021 Nov 22;12:739971. doi: 10.3389/fpls.2021.739971 (PMC8645994; doi:10.3389/fpls.2021.739971)
Supplement: Supplementary file 4 [file Table_2.DOCX]

**Table S2. The primers used to construct RNAi constructs.**

| **Primer name** | **Primer sequence (from 5'to 3')** | **Purpose** |
| --- | --- | --- |
| attB1-S1-forward primer | GGGGACAAGTTTGTACAAAAAAGCAGGCTTCACCGTGGAGTTGTGGATAAGTAA | S1 BP recombination reaction |
| attB2-S1-reverse primer | GGGGACCACTTTGTACAAGAAAGCTGGGTTCATGAAGCACACACTCGCAA |  |
| attB1-S2-forward primer | GGGGACAAGTTTGTACAAAAAAGCAGGCTTCACCGTGGCTATCTTCACTTTAGTGG | S2 BP recombination reaction |
| attB2-S2-reverse primer | GGGGACCACTTTGTACAAGAAAGCTGGGTTTCCCCAATCATGTCTTTCCTTA |  |
| attB1-S3-forward primer | GGGGACAAGTTTGTACAAAAAAGCAGGCTTCACCGAGTTCAAAATATCAAAGCTTG | S3 BP recombination reaction |
| attB2-S3-reverse primer | GGGGACCACTTTGTACAAGAAAGCTGGGTTTCAAAACTGTCCATTCCAGAGAA |  |
| attB1-S4-forward primer | GGGGACAAGTTTGTACAAAAAAGCAGGCTTCACCAGAGCTTATGCATCGCACTGA | S4 BP recombination reaction |
| attB2-S4-reverse primer | GGGGACCACTTTGTACAAGAAAGCTGGGTTTGGATCCTTACCTGCATCCAT |  |
| attB1-S5-forward primer | GGGGACAAGTTTGTACAAAAAAGCAGGCTTCACCGTGTGGGTGATGATGGATGGAG | S5 BP recombination reaction |
| attB2-S5-reverse primer | GGGGACCACTTTGTACAAGAAAGCTGGGTTTGCCTTTCAGTATTTTCGGAGTT |  |
