# Supplementary material for: Optimizing RNAi-Target by Nicotiana benthamiana-Soybean Mosaic Virus System Drives Broad Resistance to Soybean Mosaic Virus in Soybean
Source: Front Plant Sci. 2021 Nov 22;12:739971. doi: 10.3389/fpls.2021.739971 (PMC8645994; doi:10.3389/fpls.2021.739971)
Supplement: Supplementary file 5 [file Table_3.DOCX]

**Table S3. A list of oligonucleotides used for qRT-PCR, RT-PCR and genomic PCR.**

| **Primer name of qRT-PCR** | **Primer sequence (5'to 3')** | **Product size** |
| --- | --- | --- |
| JHA125-forward primer | GACAGAAAAGTAGGCCGTGAG | 118 bp |
| JHA125-reverse primer | GACCCATCCCTTTTGTACGAG |  |
| JHA17-forward primer | CACCACGCAGCTCAAGATAC | 119 bp |
| JHA17-reverse primer | GTTCCTCACGCTTTGGTTGTC |  |
| *EF1a*-forward primer | AGCTTTACCTCCCAAGTCATC | 135 bp |
| *EF1a* -reverse primer | AGAACGCCTGTCAATCTTGG |  |
| *PP2A*-forward primer | GACCCTGATGTTGATGTTCGCT | 123 bp |
| *PP2A*-reverse primer | GAGGGATTTGAAGAGAGATTTC |  |
| **RT-PCR** | | |
| pSMV-*GUS*-viral fragment(*CP*)-forward primer | TGACAATGGCACATCTCCAG | 320 bp |
| pSMV-*GUS*-viral fragment(*CP*)-reverse primer | CCGAGAGAGCTGCAGCCTTCA |  |
| *GUS*-forward primer | ATGTTACGTCCTGTAGAAACCCCA | 1812 bp |
| *GUS*-reverse primer | TCATTGTTTGCCTCCCTGCT |  |
| **genomic PCR** | | |
| *S1*-forward primer(genotype) | TAGCATGGCCGCGGGATA | 492 bp |
| *S1*-reverse primer(genotype) | AGTTTCCTGAAGATGCGCTG |  |
| *bar*-forward primer | TTCAGCAGGTGGGTGTAGA | 399 bp |
| *bar*-reverse primer | AAATTCCCCTCGGTATCCAA |  |
| *Tubulin*-forward primer | CGTTTCACCTTTGAGTCACGG | 557 bp |
| *Tubulin*-reverse primer | TGCCATGTTCGAGGCAGTAG |  |
