# Supplementary material for: Optimizing RNAi-Target by Nicotiana benthamiana-Soybean Mosaic Virus System Drives Broad Resistance to Soybean Mosaic Virus in Soybean
Source: Front Plant Sci. 2021 Nov 22;12:739971. doi: 10.3389/fpls.2021.739971 (PMC8645994; doi:10.3389/fpls.2021.739971)
Supplement: Supplementary file 6 [file Table_4.DOCX]

**Table S4. Resistance performance of the non-transgenic and transgenic soybean plants**

| SMV-tested  line | SMV strain | | | | | | | | | | |
| --- | --- | --- | --- | --- | --- | --- | --- | --- | --- | --- | --- |
|  | SC1 | SC2 | SC3 | SC4 | SC6 | SC10 | SC13 | SC16 | SC17 | SC18 | SC19 |
| WT | S | S | S | S | S | S | S | S | S | S | S |
| L6 | R | R | R | R | R | R | R | R | R | R | R |
| L7 | R | R | R | R | R | R | R | R | R | R | R |
| L10 | R | R | R | R | R | R | R | R | R | R | R |
| L12 | R | R | R | R | R | R | R | R | R | R | R |

R: resistance to SMV, indicating no visible symptoms appeared on soybean plants.

S: susceptible to SMV, indicating plants with symptoms as susceptible controls.

The transgenic lines L6, L7, L10 and L12 are homozygous T_3_ progeny and six individuals for each line are used for testing each SMV strain.

WT: wild type (receptor soybean variety NN1138-2)
