# Supplementary material for: Optimizing RNAi-Target by Nicotiana benthamiana-Soybean Mosaic Virus System Drives Broad Resistance to Soybean Mosaic Virus in Soybean
Source: Front Plant Sci. 2021 Nov 22;12:739971. doi: 10.3389/fpls.2021.739971 (PMC8645994; doi:10.3389/fpls.2021.739971)
Supplement: Supplementary file 7 [file Table_5.DOCX]

**Table S5. The similarity analysis of *S1* among the complete genome sequences of SMV strains or isolates from different countries using blastn in NCBI database.**

| **Strain or isolate name** | **GenBank accession no.** | **Country of origin** | **Collecting host** | **S1 nucleotides similarity search** | | | |
| --- | --- | --- | --- | --- | --- | --- | --- |
|  |  |  |  | **Identity (%)** | **Alignment length** | **Mismatch** | **Gap opens** |
| Am | KC845322.1 | China | *Atractylodes macrocephala koidz* | 82.3 | 464 | 76 | 6 |
| NN | KF982784.1 | China | *Pinellia pedatisecta* | 88.3 | 462 | 52 | 2 |
| LJZ010 | KP710866.1 | China | *Glycine max* | 91.8 | 462 | 38 | 0 |
| JSJJ001 | KX834321.1 | China | *Glycine soja* | 91.8 | 463 | 36 | 2 |
| BYX006 | KP710861.1 | China | *Glycine max* | 92.0 | 462 | 37 | 0 |
| 4547/CHN/2004 | HQ396725.1 | China | *Glycine max* | 92.2 | 462 | 36 | 0 |
| 4278-1 | KT285170.1 | China | *N. benthamiana* | 92.4 | 462 | 35 | 0 |
| GXQZ001 | KX834322.1 | China | *Glycine soja* | 92.9 | 462 | 33 | 0 |
| HLJHLQF001 | KX834325.1 | China | *Glycine soja* | 93.9 | 462 | 28 | 0 |
| XFQ014 | KP710876.1 | China | *Glycine max* | 94.2 | 462 | 27 | 0 |
| SX-Z | KP710870.1 | China | *Glycine max* | 94.4 | 462 | 26 | 0 |
| HLJSB001 | KX834323.1 | China | *Glycine soja* | 94.8 | 462 | 24 | 0 |
| 6202-2 | JF833014.1 | China | *Glycine max* | 95.0 | 462 | 23 | 0 |
| 6067-1 | JF833015.1 | China | *Glycine max* | 95.7 | 462 | 20 | 0 |
| Liaoning | MK350280.1 | China | *Glycine max* | 96.3 | 462 | 17 | 0 |
| SX | KC845321.1 | China | *Atractylodes macrocephala koidz* | 96.5 | 462 | 16 | 0 |
| HB-RS | KR065437.1 | China | *Glycine max* | 97.0 | 462 | 14 | 0 |
| XFQ001 | KP710871.1 | China | *Glycine max* | 97.8 | 462 | 10 | 0 |
| NE-N1 | KP710869.1 | China | *Glycine max* | 98.3 | 462 | 8 | 0 |
| SC3 | JF833013.1 | China | *Glycine max* | 99.6 | 462 | 2 | 0 |
| Sc6 | HM590054.1 | China | *Glycine max* | 99.8 | 462 | 1 | 0 |
| G1 | FJ640977.1 | Korea | *Glycine max* | 92.0 | 462 | 37 | 0 |
| G7A | FJ640982.1 | Korea | *Glycine max* | 92.4 | 462 | 35 | 0 |
| WS116 | FJ640961.1 | Korea | *Glycine soja* | 93.1 | 462 | 32 | 0 |
|  | KX096578.1 | Korea | *Glycine max* | 93.3 | 462 | 31 | 0 |
| WS155 | FJ640970.1 | Korea | *Glycine soja* | 93.5 | 462 | 30 | 0 |
| WS110 | FJ640960.1 | Korea | *Glycine soja* | 93.7 | 462 | 29 | 0 |
| G4 | FJ640979.1 | Korea | *Glycine max* | 93.9 | 462 | 28 | 0 |
| CC2515 | KY986929.1 | Korea | *Vigna angularis* | 94.2 | 462 | 27 | 0 |
| WS209 | FJ640976.1 | Korea | *Glycine soja* | 94.4 | 462 | 26 | 0 |
| WS202 | FJ640974.1 | Korea | *Glycine soja* | 94.4 | 463 | 24 | 2 |
| G7H | AY294045.1 | Korea | *Glycine max* | 94.6 | 462 | 25 | 0 |
| G5 | AY294044.1 | Korea | *Glycine max* | 94.8 | 462 | 24 | 0 |
| WS200 | FJ548849.1 | Korea | *Glycine soja* | 95.0 | 462 | 23 | 0 |
| WS101 | FJ640957.1 | Korea | *Glycine soja* | 95.2 | 462 | 22 | 0 |
| G6 | FJ640980.1 | Korea | *Glycine max* | 95.5 | 462 | 21 | 0 |
| WS32 | FJ640954.1 | Korea | *Glycine soja* | 95.7 | 462 | 20 | 0 |
| G7d | AY216987.1 | USA | *Glycine max* | 92.0 | 461 | 37 | 0 |
| G7 | AY216010.1 | USA | *Glycine max* | 92.6 | 462 | 34 | 0 |
| G2 | S42280.1 | USA | *Glycine max* | 94.2 | 462 | 27 | 0 |
| N | D00507.2 | USA | *Glycine max* | 94.4 | 462 | 26 | 0 |
| TNP | HQ845735.1 | USA | *Glycine max* | 94.6 | 462 | 25 | 0 |
| 413 | GU015011.1 | USA | *Glycine max* | 97.8 | 462 | 10 | 0 |
| BSB1 | MN124783.1 | Brasil | *passion fruit* | 93.7 | 462 | 29 | 0 |
| Rsv4-RB3 | JN416770.1 | Canada | *Glycine max* | 92.6 | 462 | 34 | 0 |
| L-RB | EU871725.1 | Canada | *Glycine max* | 93.9 | 462 | 28 | 0 |
| L | EU871724.1 | Canada | *Glycine max* | 94.4 | 462 | 26 | 0 |
| NP-C-L | HQ166265.1 | Canada | *Glycine max* | 94.6 | 462 | 25 | 0 |
| Gulupa | KY249378.1 | Colombia | *passion fruit* | 93.1 | 462 | 32 | 0 |
| India | KM979229.1 | India | unknown | 98.7 | 462 | 6 | 0 |
| Go11 | KF135491.1 | Iran | *Glycine max* | 93.5 | 462 | 30 | 0 |
| Lo3 | KF135490.1 | Iran | *Glycine max* | 94.4 | 462 | 26 | 0 |
| Ar13 | KF135488.1 | Iran | *Glycine max* | 97.6 | 462 | 11 | 0 |
| Aa15-M2 | AB100443.1 | Japan | unknown | 93.9 | 462 | 28 | 0 |
| SMV-C | LC323107.1 | Japan | *Glycine max* | 94.2 | 462 | 27 | 0 |
| P | AJ507388.2 | UK | *Pinellia ternata* | 82.8 | 464 | 74 | 5 |
| HZ1 | AJ628750.1 | UK | *aroid plants* | 87.7 | 462 | 55 | 2 |
| severe | AJ312439.1 | UK | *Pinellia ternata* | 95.0 | 462 | 23 | 0 |

The source of genomic sequences of 58 SMV strains or isolates obtained from the NCBI database (<http://www.ncbi.nlm.nih.gov/>) according to the GenBank ID.

Gap opens: numbers of gaps
